# Supplementary material for: Single-cell profiling identifies a CD8bright CD244bright Natural Killer cell subset that reflects disease activity in HLA-A29-positive birdshot chorioretinopathy
Source: Nat Commun. 2024 Jul 31;15:6443. doi: 10.1038/s41467-024-50472-0 (PMC11291632; doi:10.1038/s41467-024-50472-0)
Supplement: Supplementary file 1 — Supplementary Information [file 41467_2024_50472_MOESM1_ESM.pdf]

Supplementary information for

Single-cell profiling identifies a CD8^{bright} CD244^{bright} Natural Killer cell subset that reflects disease activity in HLA-A29-positive *birdshot chorioretinopathy*

Pulak R. Nath[1,6]*, Mary Maclean[1,7], Vijay Nagarajan[1,2], Jung Wha Lee[1], Mehmet Yakin[1], Aman Kumar[1], Hadi Nadali[1], Brian Schmidt[3], Koray D. Kaya[4], Shilpa Kodati[1], Alice Young[3], Rachel R. Caspi[2], Jonas J. W. Kuiper[5]*↾, H. Nida Sen[1]↾

[1] Clinical and Translational Immunology Unit, Laboratory of Immunology, NEI, NIH, Bethesda, USA.
[2] Immunoregulation Section, Laboratory of Immunology, NEI, NIH, Bethesda, USA.
[3] NIH Intramural Sequencing Center, NIH, Rockville, USA.
[4] Medical Genetics and Ophthalmic Genomics Unit, NEI, NIH, Bethesda, USA.
[5] Department of Ophthalmology, University Medical Center Utrecht, University of Utrecht, Utrecht, Netherlands.

[6]Current affiliation: Lentigen Technology Inc, A Miltenyi Biotec Company, 910 Clopper Road, Gaithersburg, MD 20878, USA
[7]Current affiliation: Translational Immunology Section, Office of Science and Technology, NIAMS, NIH, Bethesda, USA

* Correspondence to Pulak R. Nath (email: hellopran2000@gmail.com) and Jonas J.W. Kuiper (email: j.j.w.kuiper@umcutrecht.nl)

↾These authors jointly supervised this work.

A

Laser	Filter	Flurophore	Markers	
			Lymphocytes	Monocytes/DCs
Blue 488	695/40	PerCPCy5.5	CD3	
	550/30	FITC	CD4	
Red 628	780/60	APC-Fire 750		CD14
	730/45	AF700	CD16	CD16
	670/30	APC/A647	CD8	CD11c
UV 355	450/50	NUV450	L/D	L/D
Violet 405	660/20	BV650		HLA-DR
	610/20	BV605	CD20	CD1c
	525/50	BV510	CD56	
	450/40	BV421	CD19	CD123
Light Green 552	780/60	PeCy7		Lin

B

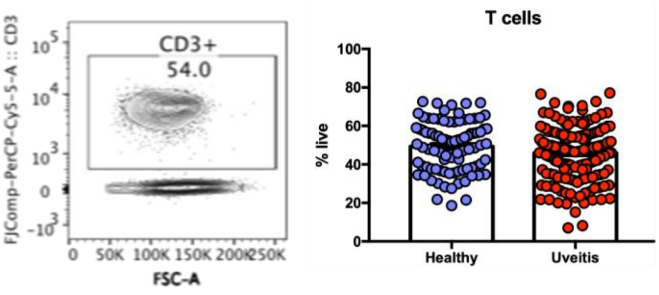

C

D

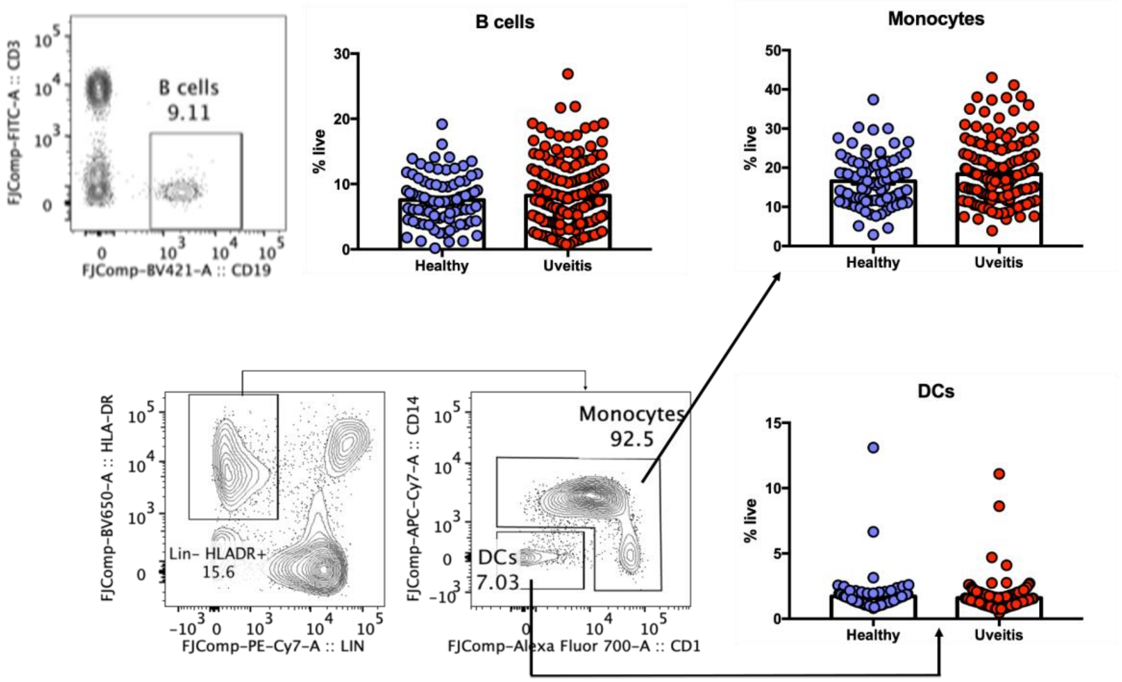

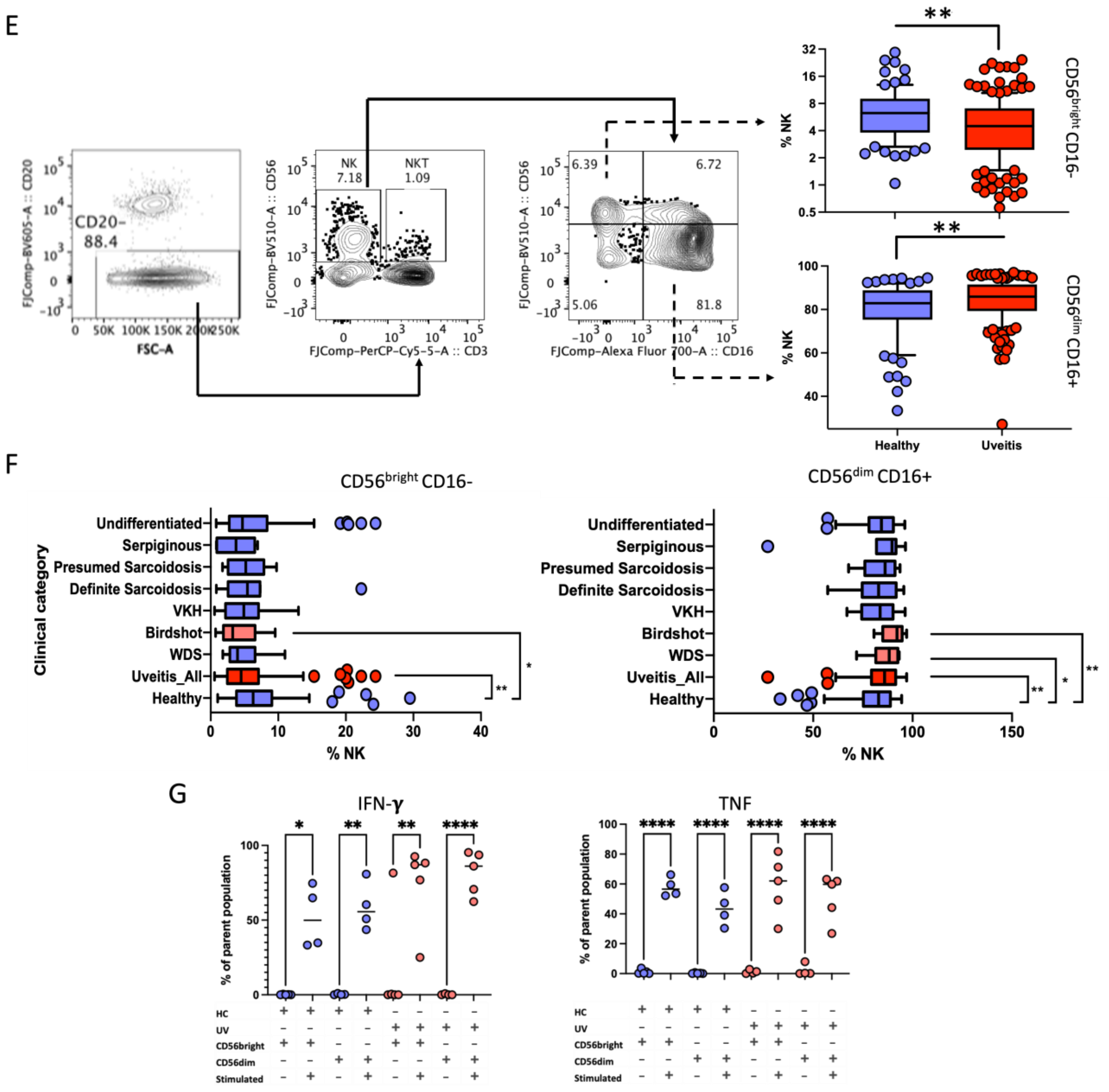

Supplementary Fig.1

A. List of markers for interrogating major lineages of immune cells (10 markers, 6 major immune subsets) in peripheral blood of patients and healthy controls using flow cytometry. Lin (Lineage) includes markers for T cells (CD3), B cells (CD19) and NK cells (CD56).

Representative plot and frequency of **(B)** T cells, **(C)** B cells, **(D)** monocyte and (DCs) and **(E)** NK cells and in the peripheral blood of uveitis patients (blue) and healthy controls are shown. There was no change in the frequency of T cells, B cells, DCs and monocytes, except the differences found in the NK cell subsets between the two groups. $CD56^{bright}CD16^{-}$ NK cells were significantly decreased while concomitantly $CD56^{dim}CD16^{+}$ NK cells were significantly increased in uveitis cohort compared to the healthy controls. Healthy *n* = 80; Uveitis *n* = 139. ** *P* = 0.002. Data are presented as mean values +/- SEM. Source data are provided as a Source Data file.

F. Flow-cytometry analysis of $CD56^{dim}$ $CD16^{+}$ and $CD56^{bright}CD16^{-}$ NK cells ($CD3^{-}CD19^{-}CD56^{+}$) in the fresh blood of different uveitis subgroups. Values are presented in the form of box and whiskers plot and represent medians with ranges (Whiskers: Tukey). WDS *n* = 18; Birdshot *n* = 18; VKH *n* = 15; Definite

sarcoidosis *n* = 8; Presumed sarcoidosis *n* = 8; Serpiginous *n* = 7; Undifferentiated *n* = 65. *P* values are from non-parametric Mann Whitney U test, ** *P* = 0.004, * *P* = 0.02. WDS, White Dot Syndromes; VKH, Vogt-Koyanagi-Harada disease.

G. IFN-γ and TNF production by $CD56^{bright}$ and $CD56^{dim}$ populations in healthy controls (HC, n= 5) vs birdshot uveitis patients (UV, n= 5) after stimulation with a leukocyte activation cocktail (eBioscience # 00-4970-03, 1/500 vol/vol). *, *P* = 0.02; **, *P* = 0.001; ***, *P* = 0.0001; ****, *P* < 0.0001. Data are presented as a line indicating the mean values. Source data are provided as a Source Data file.

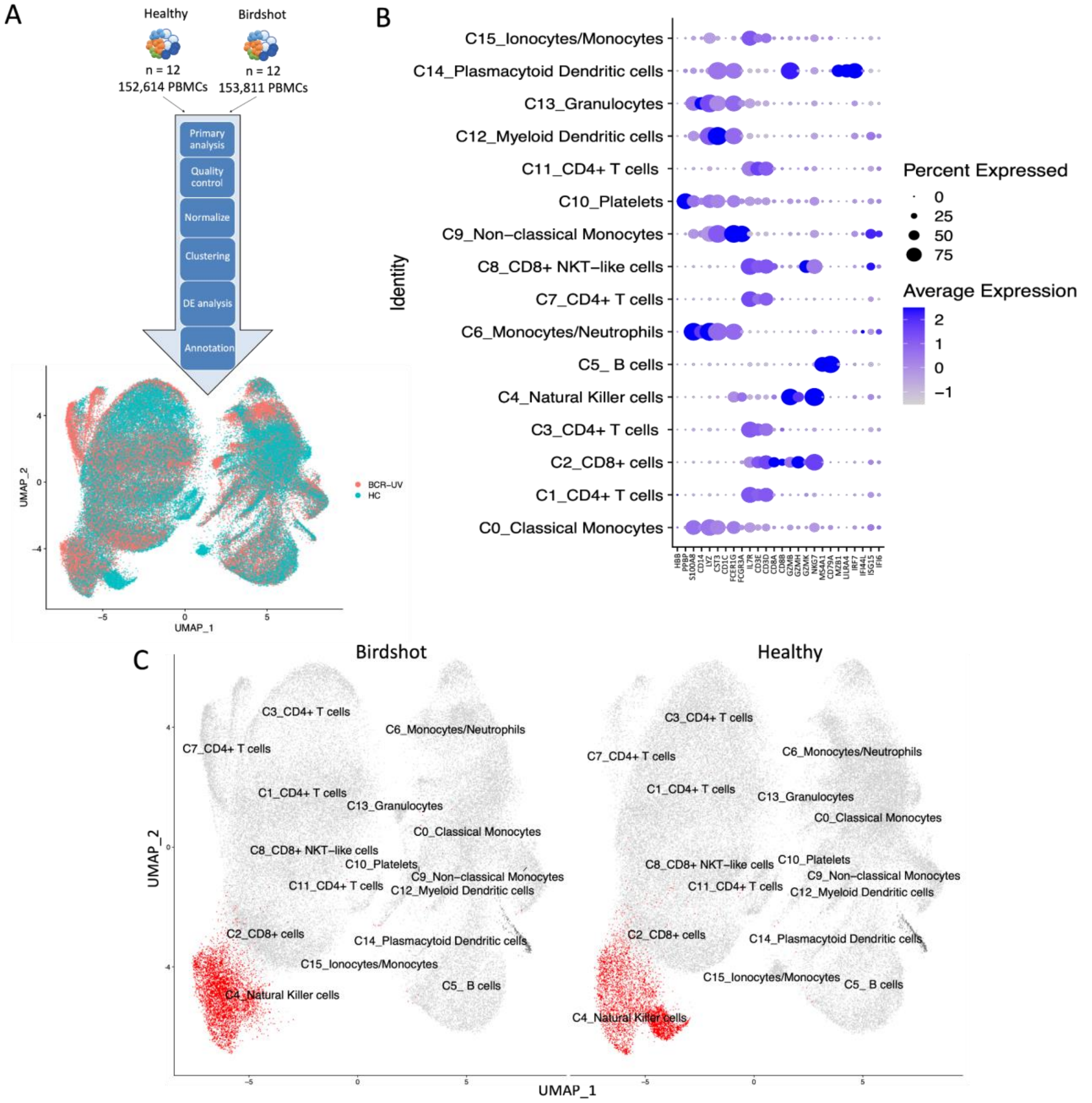
A
Healthy
Birdshot
n = 12
152,614 PBMCs
n = 12
153,811 PBMCs
Primary analysis
Quality control
Normalize
Clustering
DE analysis
Annotation
UMAP_2
UMAP_1
BCR-UV
HC
B
C15_Ionocytes/Monocytes
C14_Plasmacytoid Dendritic cells
C13_Granulocytes
C12_Myeloid Dendritic cells
C11_CD4+ T cells
C10_Platelets
C9_Non−classical Monocytes
C8_CD8+ NKT−like cells
C7_CD4+ T cells
C6_Monocytes/Neutrophils
C5_ B cells
C4_Natural Killer cells
C3_CD4+ T cells
C2_CD8+ cells
C1_CD4+ T cells
C0_Classical Monocytes
Identity
Percent Expressed
0
25
50
75
Average Expression
2
1
0
−1
C
Birdshot
Healthy
UMAP_2
UMAP_1

D

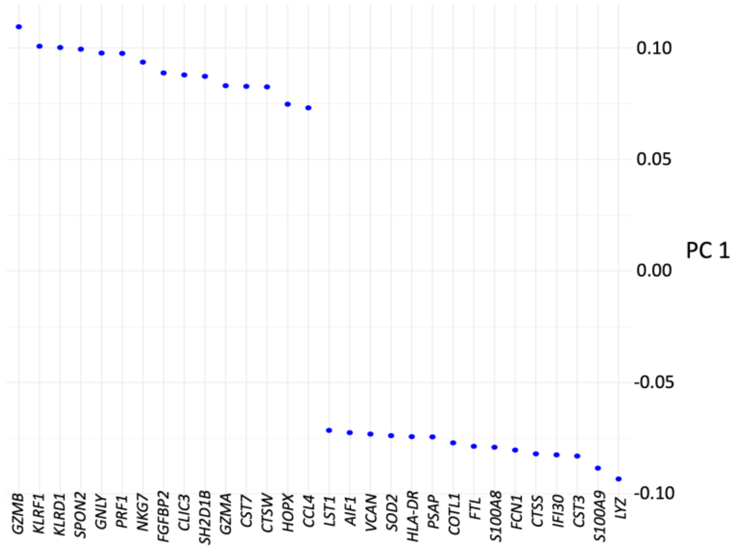

Supplementary Fig. 2
A. An overview of scRNAse analysis. A total of 306,425 PBMCs from 12 birdshot uveitis patients (Birdshot/BCR-UV) and 12 healthy controls (Healthy) were subjected to 10x Chromium single cell separation and sequencing and merged to a combined dataset. The bioinformatic analysis pipeline includes the Primary analysis, Quality control, Normalization, Clustering, Differential Expression (DE) analysis and Annotation of the combined dataset. The UMAP plot (top) shows the merged distribution of cells from Healthy (Green) and BCR-UV (Red). The UMAP plot (bottom) shows annotation of 16 clusters of cell lineages in the combined dataset. The putative identity of each cluster was manually annotated based on 'scsa'[1] and 'scType'[2] annotations and interrogating the top expressed genes in each cluster.
B. Dot plot showing the expression profile of lineage-specific genes in the annotated cell clusters.
C. The UMAP plot of single cell distribution is representing 16 clusters of cell lineages in the combined dataset of a total of 306,425 analyzed cells. The putative identity of each cluster was manually annotated. The NK cell clusters are identified in red color and the remaining cell clusters are presented in grey color.
D. The top 30 most differentially expressed NK genes defined by principal component 1 in BCR-UV compared to HC.

A

Cluster	HC	BCR-UV
0	974	1119
1	948	275
2	351	862
3	508	482
4	478	453
5	275	432
6	208	457
7	201	324
8	185	323
9	206	241
10	25	235
11	26	31

B

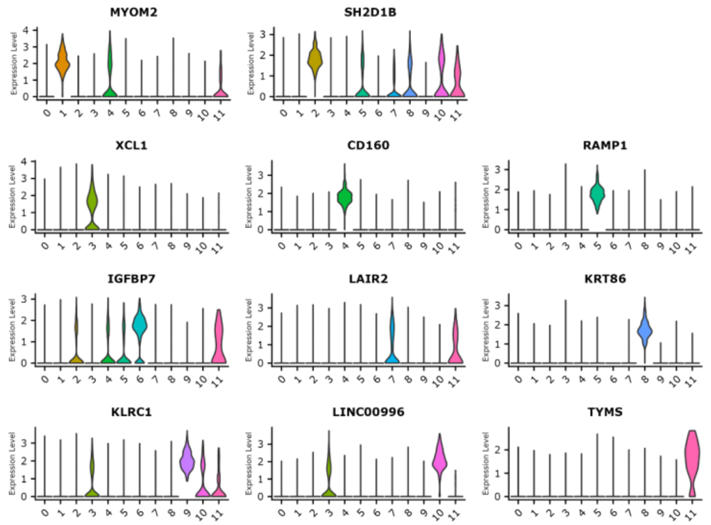

C

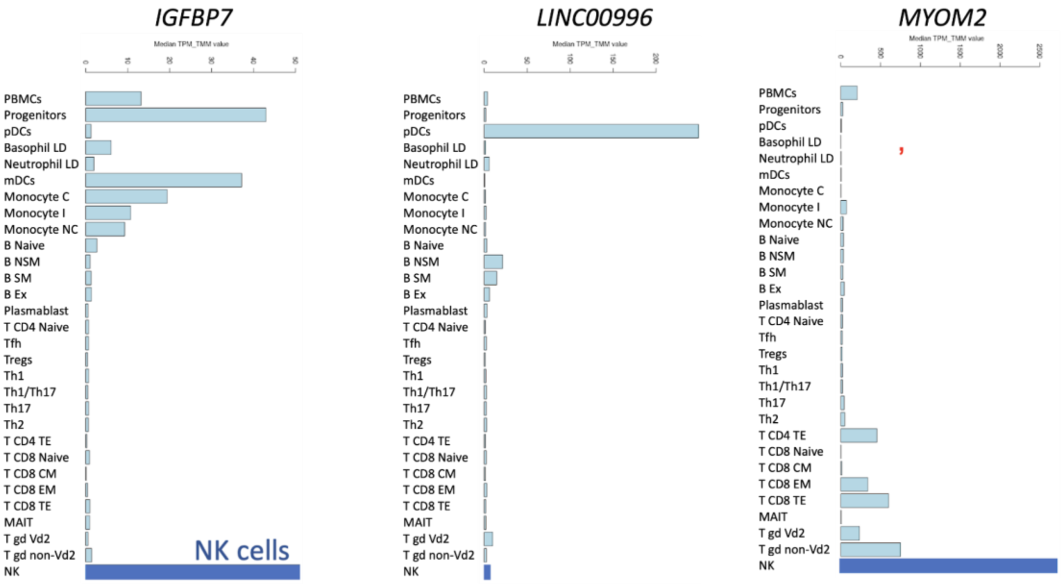

D

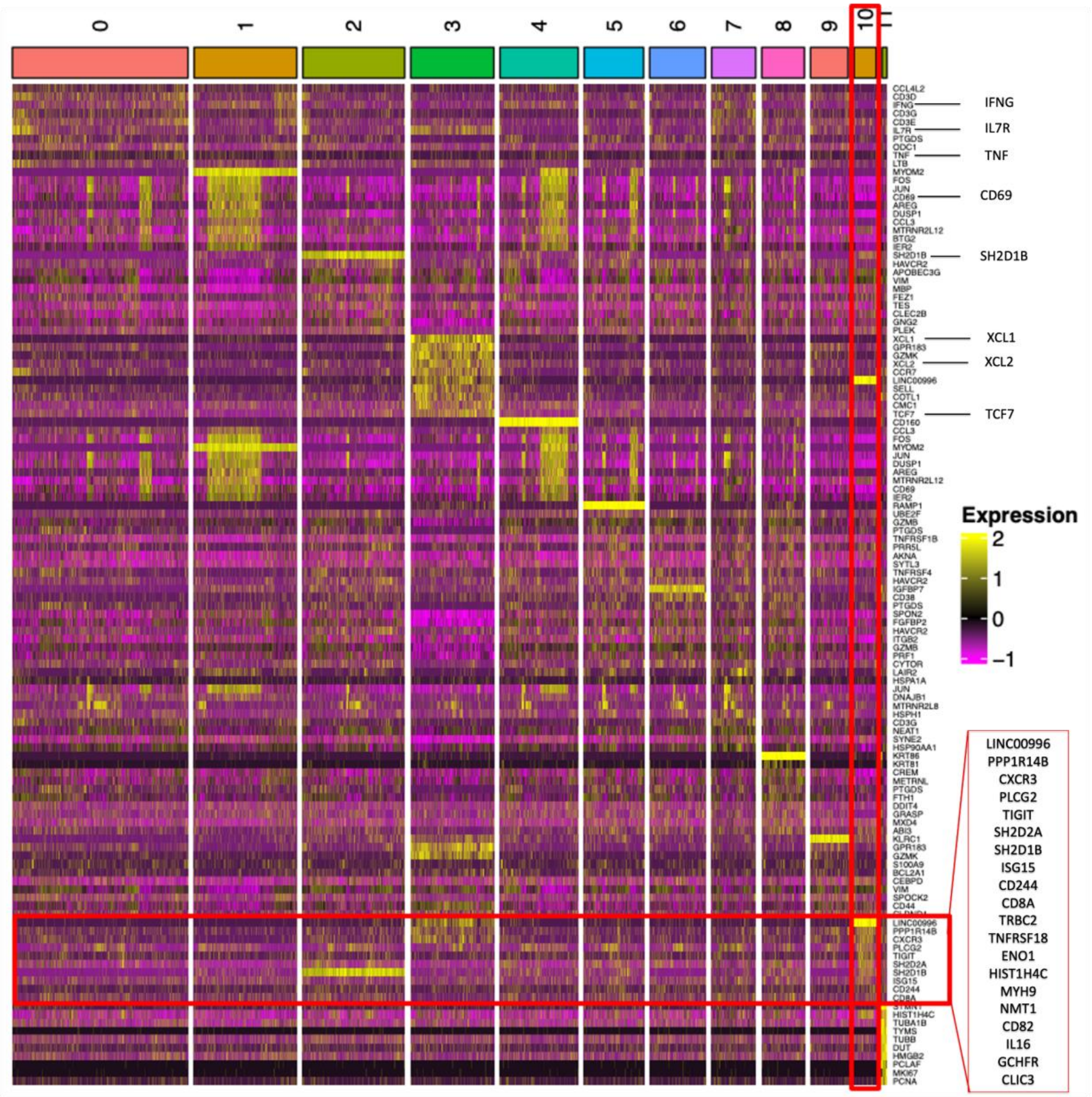

Supplementary Fig. 3

A. Tabular representation of total 12 NK-subclusters and associated cell count in 12 healthy controls (HC) and in 12 birdshot uveitis patients (BCR-UV).

B. Violin plots representing the list of genes that are uniquely expressed in each of the 12 clusters of NK cell-subclusters.

C. Expression check for *IGFBP7*, *LINC00996* and *MYOM2* genes across several peripheral blood immune cell populations (ABIS database[3]).

D. Heatmap representing the expression of top 10 highly expressed markers of each NK cluster.

A

Laser	Filter	Fluorophore	Markers
Blue 488	695/40	PerCPCy5.5	CD3
	550/30	FITC	CD94
Red 628	780/60	APC-Fire 750	CD8
	730/45	AF700	CD16
	670/30	APC/A647	CD57
UV 355	450/50	NUV450	L/D
Violet 405	660/20	BV650	CD314
	610/20	BV605	CD20
	525/50	BV510	CD56
	450/40	BV421	CD158e1
Light Green 552	780/60	PeCy7	CD337
	610/20	PE-Dazzle	CD244
	582/15	PE	CD336

B

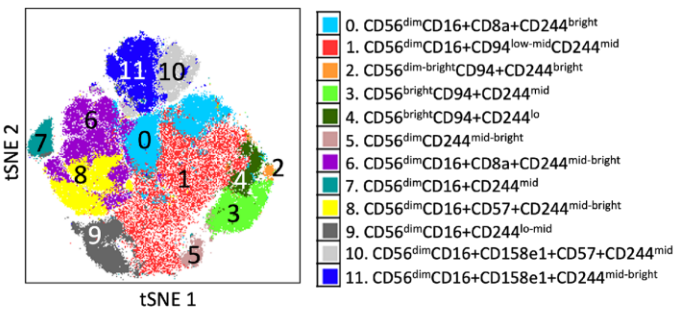

C

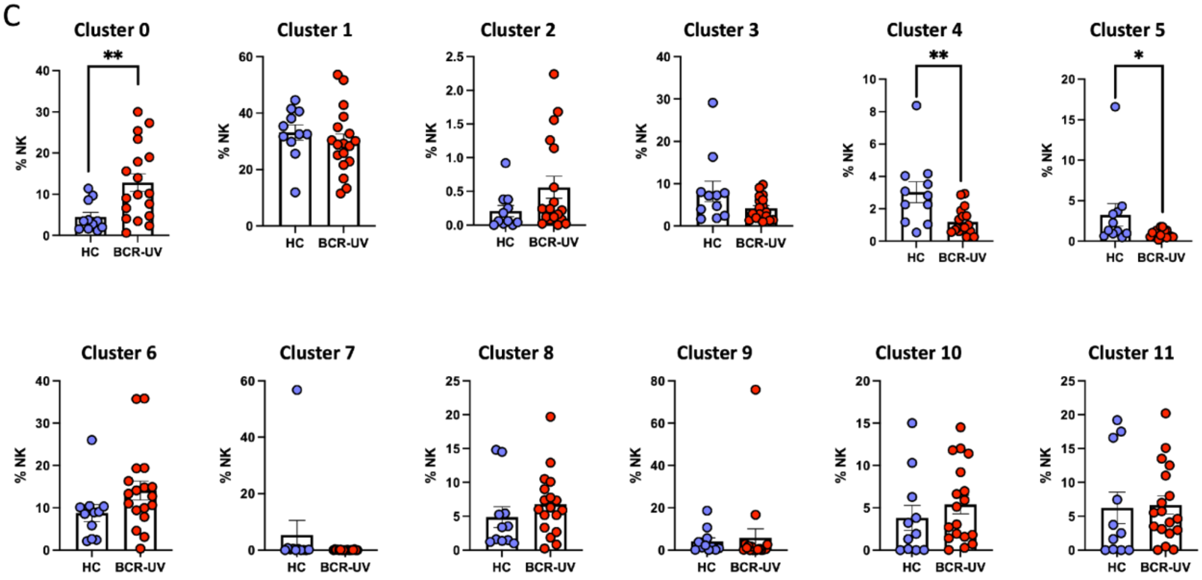

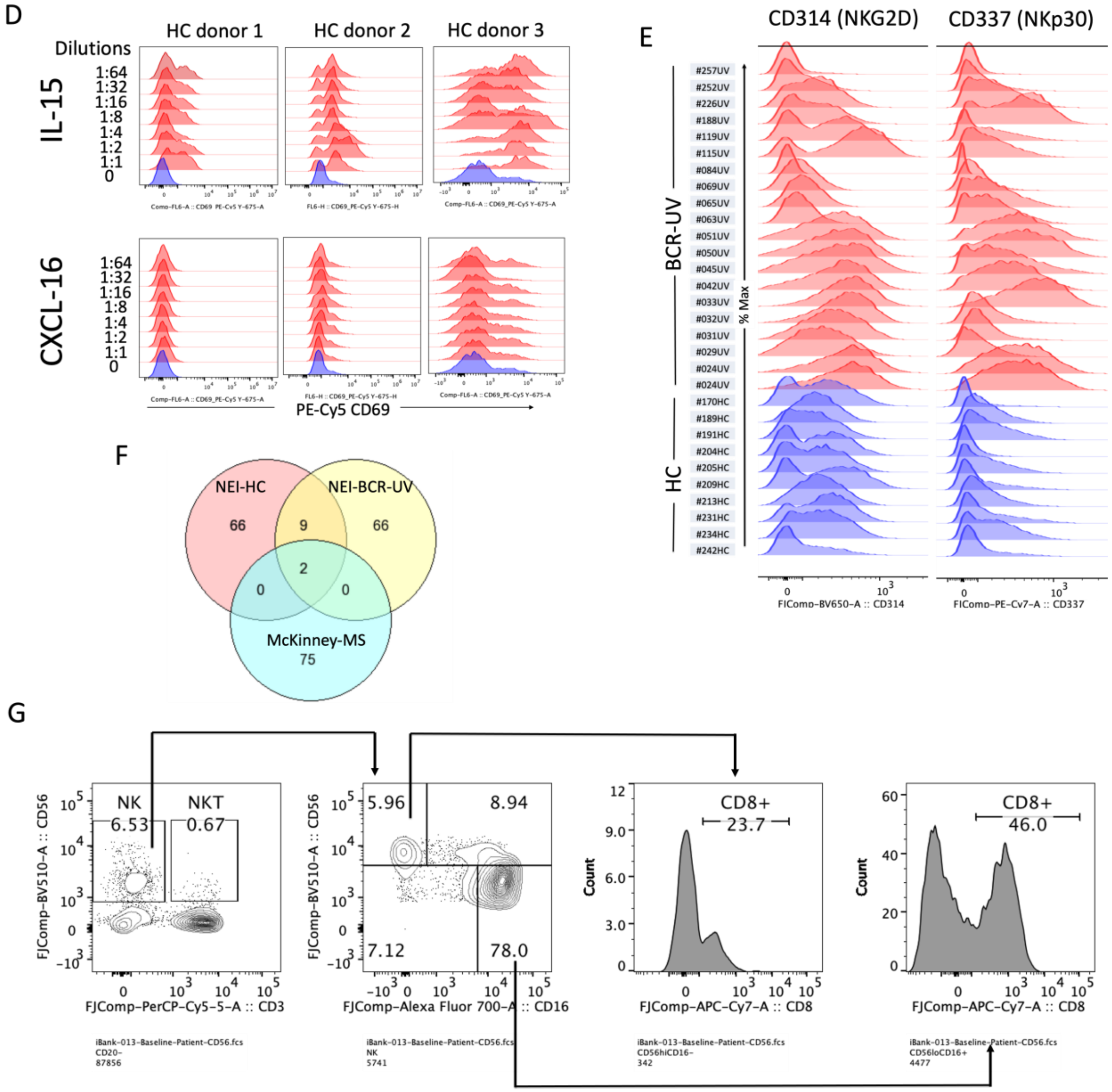

Supplementary Fig. 4

A. List of markers used to interrogate NK lineage cells in peripheral blood of BCR-UV patients and healthy controls using flow cytometry.

B. t-SNE plot represents the FlowJo-based FlowSOM analysis to identify clusters based on NK cell surface markers from a combined dataset of healthy controls and birdshot uveitis patients. Cluster 0 ($CD56^{dim}CD16^{+}CD8^{+}$ and $CD244^{bright}$) is one out of 12 NK clusters that was elevated in birdshot uveitis. Phenotypes of all 12 NK clusters are color coded and described. Healthy n = 11, Birdshot n = 18.

C. Scatter plot of the frequency of cells of clusters 0 to 11 in peripheral blood in BCR-UV patients (BCR-UV, red dots) versus healthy controls (HC, blue dots). HC n = 10, BCR-UV n = 18. *, P = 0.02; **, P = 0.001. Data are presented as mean values +/- SEM. Source data are provided as a Source Data file.

D. Histograms showing the surface expression of CD69 within the $CD8a^{+}$ NK cell compartment of peripheral blood isolated from three healthy donors and stimulated for 48 hrs with indicated dilutions of recombinant human IL-15 and CXCL-16 proteins. Blue peaks represent untreated cells and red peaks represent cytokine-treated cells.

E. Histograms showing expression of CD314 (NKG2D) and CD337 (NKp30) in NK cells of 10 healthy controls (blue) and 18 birdshot uveitis (red).

F. Comparing top 77 genes expressed in CD8+ NK cells in BCR-UV patients (BCR-UV), healthy control (HC) and recently described NK8+ population in multiple sclerosis patients[4] (McKinney-MS).

G. Flow cytometry gating strategy to evaluate CD8 expression profiles in $CD56^{bright}$ and $CD56^{dim}CD16^{+}$ subsets of NK cells.

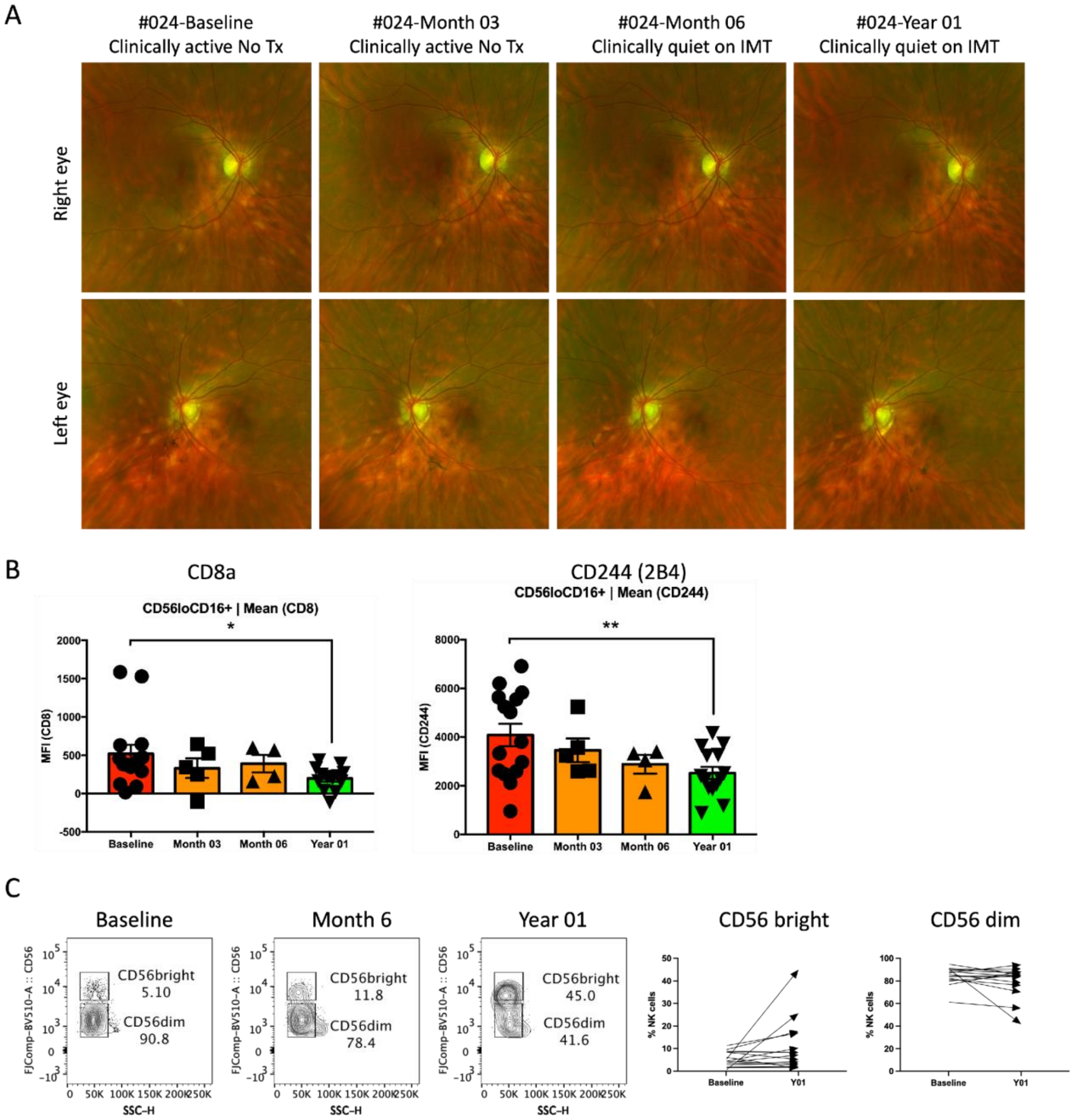

Supplementary Fig. 5

A. Fundoscopy examination of both eyes of a birdshot uveitis patient (#024) at the Baseline, after Month 03, Month 06 and Year 01 of systemic immunomodulatory treatment (IMT).

B. Bar plots indicate statistical significance on changes of CD8a and CD244 MFI within $CD56^{dim}CD16^{+}$ NK cells from 15 different birdshot uveitis patients from baseline to year 01 after treatment. Baseline, *n* = 15; Month 03, *n* = 4; Month 06, *n* = 4 and Year 01, *n* = 15. Statistical comparison is done using the

unpaired t-test between baseline and year 01. *, $P = 0.01$; **, $P = 0.007$. Data are presented as mean values +/- SEM. Source data are provided as a Source Data file.
C. Changes in CD56$^{bright/dim}$ NK cells populations after treatment are evaluated by flow cytometry.

Supplementary References

1. Cao, Y. *et al.* SCSA: A Cell Type Annotation Tool for Single-Cell RNA-seq Data. *Front Genet* 11, 490 (2020).
2. Ianevski, A. *et al.* Fully-automated and ultra-fast cell-type identification using specific marker combinations from single-cell transcriptomic data. *Nat Commun* 13, 1246 (2022).
3. https://giannimonaco.shinyapps.io/ABIS/
4. McKinney, E.F. *et al.* A CD8(+) NK cell transcriptomic signature associated with clinical outcome in relapsing remitting multiple sclerosis. *Nat Commun* 12, 635 (2021).
