# Supplementary material for: Single-cell profiling identifies a CD8bright CD244bright Natural Killer cell subset that reflects disease activity in HLA-A29-positive birdshot chorioretinopathy
Source: Nat Commun. 2024 Jul 31;15:6443. doi: 10.1038/s41467-024-50472-0 (PMC11291632; doi:10.1038/s41467-024-50472-0)
Supplement: Supplementary file 3 — Description of Additional Supplementary Files [file 41467_2024_50472_MOESM3_ESM.pdf]

Description of Additional Supplementary Files

Title: Supplementary Data 1

Description: The table shows the donor demography of 80 healthy control and 139 uveitis patients. The demography includes age, gender, flow cytometric staining and acquisition dates.

Title: Supplementary Data 2

Description: List of the antibodies used for flow cytometric staining. The table includes antibody specificity, fluorochromes, clone number, catalog number, vendor and dilutions used for staining.

Title: Supplementary Data 3

Description: List of 12 healthy and 12 birdshot uveitis patients PBMC samples used for single cell RNAseq (scRNAseq). The table includes sample batch, sample preparation date and date when data was received.

Title: Supplementary Data 4

Description: List of 10x Genomics reagents including primers and barcodes used for the scRNAseq sample preparation. The primer list includes sequences of Gel Bead Primer, Template Switch Primer, cDNA Forward Primer, cDNA Reverse Primer, Adapter Primer 1, Adapter Primer 2, SI Primer P5, SI Primer P7. The Barcode list includes Barcode set SI-GA-A1-12, SI-GA-B1-12, SI-GA-C1-12, SI-GA-D1-12, SI-GA-E1-12, SI-GA-F1-12, SI-GA-G1-12 and SI-GA-H1-12 and 8 nucleotide sequences of Barcode 1-4 for each of the above Barcode set.

Title: Supplementary Data 5

Description: scRNAseq library info of 12 healthy control and 12 birdshot uveitis patients. Out of many listed in this table, some important parameters include number of cells sequenced from each donor, mean reads per cell, Median Genes per Cell, Number of Reads, Valid Barcodes,

Sequencing Saturation, Reads Mapped to Genome, Reads Mapped Confidently to Genome, Reads Mapped to Transcriptome, Total Genes Detected and Median UMI Counts per Cell.

Title: Supplementary Data 6

Description: Frequencies of NK cell subclusters from the FlowJo-based FlowSOM analysis. The table includes frequencies of each of the 12 subclusters analyzed by FlowSOM analysis from 11 healthy control and 18 birdshot uveitis patients.

Title: Supplementary Data 7

Description: Disease and treatments details of the birdshot uveitis patients. The parameters listed in the table include date of recruitment, age, sex, CME, Treatment Category, Location category, Categorical diagnosis, Clinic Activity, Angiographic Activity, Retinal vascular involvement (clinical or FA based), Systemic prednisone, Anti-metabolite, Biologic (TNFi), T-cell inhibitors (Cyclosporine, Tacrolimus, Sirolimus), Patients required biologic anytime, Number of systemic immunsuppressive medications at each visit, Specific diagnosis, Laterality, Systemic Baseline treatment and Details of IMT.
