# Supplementary material for: Single-cell profiling identifies a CD8bright CD244bright Natural Killer cell subset that reflects disease activity in HLA-A29-positive birdshot chorioretinopathy
Source: Nat Commun. 2024 Jul 31;15:6443. doi: 10.1038/s41467-024-50472-0 (PMC11291632; doi:10.1038/s41467-024-50472-0)
Supplement: Supplementary file 5 — Reporting Summary [file 41467_2024_50472_MOESM5_ESM.pdf]

nature portfolio

Corresponding author(s): Pulak Ranjan Nath, Jonas J. W. Kuiper

Last updated by author(s): 04/26/2024

# Reporting Summary

Nature Portfolio wishes to improve the reproducibility of the work that we publish. This form provides structure for consistency and transparency in reporting. For further information on Nature Portfolio policies, see our Editorial Policies and the Editorial Policy Checklist.

## Statistics

For all statistical analyses, confirm that the following items are present in the figure legend, table legend, main text, or Methods section.

| n/a | Confirmed | |
|---|---|---|
| ☐ | ☒ | The exact sample size (*n*) for each experimental group/condition, given as a discrete number and unit of measurement |
| ☒ | ☐ | A statement on whether measurements were taken from distinct samples or whether the same sample was measured repeatedly |
| ☐ | ☒ | The statistical test(s) used AND whether they are one- or two-sided<br>*Only common tests should be described solely by name; describe more complex techniques in the Methods section.* |
| ☐ | ☒ | A description of all covariates tested |
| ☒ | ☐ | A description of any assumptions or corrections, such as tests of normality and adjustment for multiple comparisons |
| ☐ | ☒ | A full description of the statistical parameters including central tendency (e.g. means) or other basic estimates (e.g. regression coefficient) AND variation (e.g. standard deviation) or associated estimates of uncertainty (e.g. confidence intervals) |
| ☐ | ☒ | For null hypothesis testing, the test statistic (e.g. *F*, *t*, *r*) with confidence intervals, effect sizes, degrees of freedom and *P* value noted<br>*Give P values as exact values whenever suitable.* |
| ☒ | ☐ | For Bayesian analysis, information on the choice of priors and Markov chain Monte Carlo settings |
| ☒ | ☐ | For hierarchical and complex designs, identification of the appropriate level for tests and full reporting of outcomes |
| ☒ | ☐ | Estimates of effect sizes (e.g. Cohen's *d*, Pearson's *r*), indicating how they were calculated |

*Our web collection on statistics for biologists contains articles on many of the points above.*

## Software and code

Policy information about availability of computer code

| | |
|---|---|
| Data collection | No software was used for data collection. |
| Data analysis | Flow cytometry data was analyzed using FlowJo v.10 software; statistical analysis of data was done using GraphPad Prism 10. Single cell data analysis was done using custom workflow scripts, written using R and BASH programming languages. The workflow components include cellranger, Seurat, SCSA, SCTYPE, Plotly, ggplot and other dependent packages. This work utilized the computational resources of the NIH HPC Biowulf cluster. Entire code, example demo data and the detailed documentation are available for public use at the github site https://github.com/PulakNath/bcr-uveitis |

For manuscripts utilizing custom algorithms or software that are central to the research but not yet described in published literature, software must be made available to editors and reviewers. We strongly encourage code deposition in a community repository (e.g. GitHub). See the Nature Portfolio guidelines for submitting code & software for further information.

## Data

Policy information about availability of data

All manuscripts must include a data availability statement. This statement should provide the following information, where applicable:

- Accession codes, unique identifiers, or web links for publicly available datasets
- A description of any restrictions on data availability
- For clinical datasets or third party data, please ensure that the statement adheres to our policy

The primary data used in this research is deposited and available for the public, without any restrictions, at the NCBI SRA repository under the accession

PRJNA855114 https://www.ncbi.nlm.nih.gov/sra/?term=PRJNA855114
Entire code, example demo data and the detailed documentation are available for public use at the github site https://github.com/PulakNath/bcr-uveitis

## Research involving human participants, their data, or biological material

Policy information about studies with human participants or human data. See also policy information about sex, gender (identity/presentation), and sexual orientation and race, ethnicity and racism.

| | |
|---|---|
| Reporting on sex and gender | Sex of the total 219 study participants were determined based on self-reports and has been reported in the Source Data with consent. There was almost equal distribution of participants of both sexes in this study. And there is no previous report of NK cell to discriminate among the genders. Therefore sex and gender were not considered in the study design. |
| Reporting on race, ethnicity, or other socially relevant groupings | This study is not based on race, ethnicity or other socially relevant groupings. |
| Population characteristics | Average age of healthy controls participated in this study was 42 (+/- 12.5 SD) and average age of uveitis patients was 44 (+/- 18 SD). Males and females were distributed equally in this study for both healthy controls and patients groups. The ethnicity of the recruited participants included white, black, Asian and hispanic origin. |
| Recruitment | Participants were recruited if they:<br>Had a diagnosis of uveitis, scleritis or a disease known to be associated with intraocular inflammation, (e.g., sarcoidosis, Behcet's disease, MS and lymphoma) OR could serve as an unaffected control.<br>Were eight years of age or older if an affected participant.<br>Were 18 years of age or older if serving as an unaffected control.<br>For participants 18 years of age and older were willing to give informed consent that includes collection and study of at least one peripheral blood sample.<br>For minor patients, their parents/Legally authorized representative were willing to give informed consent that includes collection and study of at least one peripheral blood sample.<br><br>Participants were not recruited if they:<br>Were unable to understand and sign the informed consent form.<br>Were unable or unwilling to give informed consent that includes use of medical records and clinical samples for current and future research related to vision and diseases affecting the eyes.<br>Had a systemic disease that compromises the ability to provide adequate ophthalmologic examination or treatment as determined by the investigator.<br><br>For participants with uveitis were not recruited if they:<br>Had inactive anterior uveitis or quiescent infectious uveitis not requiring such regimented and intensive standardized testing as determined by the Investigator.<br>Had end stage or chronic quiescent changes in the setting of an established infectious etiology, such as an old ocular toxoplasma scar (participants with active intraocular inflammation due to infection will be recruited).<br><br>Once consented, patients underwent screening evaluations as specified in the protocols. Participants were recruited by the investigators pursuant to IRB approved methods to reduce the possibility of biased recruiting. |
| Ethics oversight | This study was conducted in compliance with the Declaration of Helsinki and ethical principles regarding human experimentation. All samples were obtained under a National Institutes of Health (NIH) Institutional Review Board (IRB) approved protocol (Uveitis/Intraocular Inflammatory Disease Biobank (iBank); NCT02656381). Informed consent was obtained from all enrolled participants. |

Note that full information on the approval of the study protocol must also be provided in the manuscript.

# Field-specific reporting

Please select the one below that is the best fit for your research. If you are not sure, read the appropriate sections before making your selection.

☒ Life sciences ☐ Behavioural & social sciences ☐ Ecological, evolutionary & environmental sciences

For a reference copy of the document with all sections, see nature.com/documents/nr-reporting-summary-flat.pdf

# Life sciences study design

All studies must disclose on these points even when the disclosure is negative.

| | |
|---|---|
| Sample size | Total 139 adult patients (Supplementary Table 1) with non-infectious uveitis (NIU), and 80 age-, sex- and race-matched healthy donors were recruited at the National Eye institute (NEI) outpatient clinic for this study. No statistical methods were used to predetermine the sample size. The number of samples was determined based on previous research experience and actual collection conditions, and can also meet statistical requirements. |
| Data exclusions | No data from enrolled patients or healthy donors were excluded from analysis. |

| | |
|---|---|
| Replication | All in vitro experiments were independently performed at least three times successfully and representative data was presented. Sample sizes of patients and healthy donors in each figures are noted to indicate the degree of replication of the results. |
| Randomization | Patient participants in the analysis was randomized. |
| Blinding | Patients were recruited by the clinicians and samples were renamed before handing over to investigators. Samples were analyzed by investigators blinded on patient/healthy donor details. |

# Reporting for specific materials, systems and methods

We require information from authors about some types of materials, experimental systems and methods used in many studies. Here, indicate whether each material, system or method listed is relevant to your study. If you are not sure if a list item applies to your research, read the appropriate section before selecting a response.

## Materials & experimental systems

| n/a | Involved in the study |
|---|---|
| ☐ | ☒ Antibodies |
| ☒ | ☐ Eukaryotic cell lines |
| ☒ | ☐ Palaeontology and archaeology |
| ☒ | ☐ Animals and other organisms |
| ☐ | ☒ Clinical data |
| ☒ | ☐ Dual use research of concern |
| ☒ | ☐ Plants |

## Methods

| n/a | Involved in the study |
|---|---|
| ☒ | ☐ ChIP-seq |
| ☐ | ☒ Flow cytometry |
| ☒ | ☐ MRI-based neuroimaging |

## Antibodies

Antibodies used

CD3 Alexa Fluor 488, clone OKT3, Cat# 317310, Biolegend, dil 1:300
CD3 PerCP-Cy5.5, clone SK7, Cat# 344808, Biolegend, dil 1:300
CD3 Biotin- PE-Cy7, clone SK7, Cat# 344820, Biolegend, dil 1:300
CD16 Alexa Fluor 700, clone 3G8, Cat# 302026, Biolegend, dil 1:100
CD14 APC-Fire 750, clone 63D3, Cat# 367120, Biolegend, dil 1:200
CD8 APC-Fire 750, clone SK1, Cat# 344746, Biolegend, dil 1:300
CD57 APC, clone HNK-1 , Cat# 359610, Biolegend, dil 1:200
CD19 Biotin- PE-Cy7, clone HIB19, Cat# 302204, Biolegend, dil 1:300
CD19 BV421, clone HIB19, Cat# 302234, Biolegend, dil 1:300
CD20 Biotin- PE-Cy7/BV605, clone 2H7, Cat# 302350, Biolegend, dil 1:300
CD20 BV510, clone 2H7, Cat# 302340, Biolegend, dil 1:300
CD56 Biotin- PE-Cy7, clone HCD56, Cat# 318320, Biolegend, dil 1:200
CD56 BV510, clone HCD56, Cat# 318340, Biolegend, dil 1:200
CD158e1 BV421, clone DX9, Cat# 312714, Biolegend, dil 1:300
HLA-DR BV650, clone L243, Cat# 307650, Biolegend, dil 1:100
CD314 BV650, clone 1D11, Cat# 563408, BD, dil 1:50
CD94 FITC, clone DX22, Cat# 305504, Biolegend, dil 1:300
CD4 FITC, clone SK3, Cat# 344604, Biolegend, dil 1:300
CD336 PE, clone P44-8, Cat# 325108, Biolegend, dil 1:50
CD337 PE-Cy7, clone AF29-4D12, Cat# 25-3379-42, Thermo Fisher, dil 1:50
CD244 PE-Dazzle 594, clone C1.7, Cat# 329522, Biolegend, dil 1:300

Validation

All antibodies used in this study are commercially available. Antibody validations were performed y the suppliers and the information is provided on the website and product information datasheets. The certificate of analysis (CoA) was provided for the quality assurance of each antibody lot. Each antibody was titrated for flow cytometry in healthy control PBMCs and determined the appropriate dilutions for staining.

## Clinical data

Policy information about clinical studies

All manuscripts should comply with the ICMJE guidelines for publication of clinical research and a completed CONSORT checklist must be included with all submissions.

| | |
|---|---|
| Clinical trial registration | NCT02656381 |
| Study protocol | https://clinicalstudies.info.nih.gov/ProtocolDetails.aspx?id=2016-EI-0046 |
| Data collection | July 26, 2016 to May 4, 2023 |
| Outcomes | Not relevant. |

## Plants

| | |
|---|---|
| Seed stocks | Not relevant. |
| Novel plant genotypes | Not relevant. |
| Authentication | Not relevant. |

## Flow Cytometry

### Plots

Confirm that:

- [x] The axis labels state the marker and fluorochrome used (e.g. CD4-FITC).
- [x] The axis scales are clearly visible. Include numbers along axes only for bottom left plot of group (a 'group' is an analysis of identical markers).
- [x] All plots are contour plots with outliers or pseudocolor plots.
- [x] A numerical value for number of cells or percentage (with statistics) is provided.

### Methodology

| | |
|---|---|
| Sample preparation | Blood samples from patients and healthy controls were collected through venipuncture and all the samples were processed within 4h of blood collection. Three mL of fresh whole-blood samples were directly used for flow cytometry staining and acquisition. PBMCs were purified by standardized density gradient isolation (Ficoll-Paque) and stored in liquid nitrogen until further use. NK cells were isolated from PBMCs using human NK cell isolation kit (Miltenyi Biotec, Catalog no. 130-092-657). |
| Instrument | BD LSR Fortessa II |
| Software | FlowJo V.10 |
| Cell population abundance | NK cells are in the rage between 5-15% of total lymphocytes. |
| Gating strategy | For FlowJo analysis, starting cell population was the lymphocyte population gated from SSC-A/FSC-A axis followed by singlets gating from SSC-H/FSC-H axis. Aqua L/D negative cells were gated for Live cells. Next, from CD56/CD3 axis, CD56+ and CD3- population were gated as NK cells. For intracellular staining, protein expression in NK cells/ NK subsets were presented in histogram. NK cell gating strategy is provided in the supplementary figure 1. |

- [x] Tick this box to confirm that a figure exemplifying the gating strategy is provided in the Supplementary Information.
